# Longitudinal agreement of repeated critical haemoglobin measurements as evidence of sustained analytical quality

**DOI:** 10.3389/bjbs.2026.16480

**Published:** 2026-08-03

**Authors:** Vanda Simões, Cacilda Magalhães, Yuliana O. Eremina

**Affiliations:** 1 Clinical Pathology Department, Local Health Unit of Matosinhos, Matosinhos, Portugal; 2 MEDCIDS, Faculty of Medicine, University of Porto, Porto, Portugal; 3 INESC TEC, Porto, Portugal; 4 EPIUnit – Public Health Institute, University of Porto, Porto, Portugal; 5 Laboratory for Integrative and Translational Investigation in Population Health (ITR), Porto, Portugal

**Keywords:** critical haemoglobin, internal quality control, longitudinal control, raw analytical data, repeated measurements

Dear Editors,

Haemoglobin (Hb) values below 70 g/L are widely recognised as critical results and have traditionally prompted repeat analysis prior to reporting. This practice emerged as a quality assurance safeguard intended to detect potential analytical random or systematic errors before result validation, a practice whose clinical utility is increasingly being questioned [1, 2], but still exists in many laboratories.

In contemporary laboratory practice, however, highly automated haematology platforms operate with real-time flagging systems and advanced error detection algorithms. Furthermore, raw analytical data archived in analyser backups include repeated full/complete blood count (FBC/CBC) measurements performed for technical or clinical reasons, such as reflex testing, flag resolution, or platelet confirmation using alternative methods. These naturally occurring duplicate measurements generate a real-world dataset that can be analysed to monitor analytical performance and quantify imprecision under routine operating conditions [3].

We conducted a retrospective longitudinal evaluation at a secondary public hospital, at the laboratory that serves a heterogeneous patient population, receiving samples from diverse clinical settings, including the emergency department, inpatient wards, day hospital services, and primary care centres. Raw analytical blocks of data were extracted from the back-up databases of three interconnected Sysmex XN-9100 modules between 2021 and 2025. Data management, exploratory data analysis and automated sample pairing were performed using R (version 4.4.3).

A total of 499,965 haemograms were reviewed and, among these, 3,154 haemograms with Hb <70 g/L were identified. Of these, 94.8% (n = 2,990) underwent repeat analysis according to the laboratory’s standard operating procedure (SOP), while 5.2% (n = 164) were validated by a clinical pathologist without repetition based on clinical history and urgency, following a defined override process, and were therefore excluded from the analytical performance assessment. For each episode, all repetitions (2–8 per sample) were analysed to determine the maximum absolute difference (ΔHb) between the initial and repeated measurement, as we aimed to characterise the maximum discrepancy that could occur under routine conditions, providing a conservative assessment of clinical risk. Reanalysis occurred either on the same analytical module (intra-analyser) or on a different module (inter-analyser) [4].

Retesting episodes were defined as immediate reflex re-runs performed on the same EDTA tube on the Sysmex Automation Line that is configured to automatically perform such re-runs for all samples with Hb<70 g/L. Additionally, 43 samples were analysed primarily in manual mode, and 102 samples had at least one manual repeat; these were not excluded, as our aim was to evaluate the SOP implemented in our routine clinical practice.

A total of 2,990 unique samples were included in the final analysis (1,991 for intra-analyser and 999 for inter-analyser), representing 6,819 total automated measurements. The global results demonstrated high analytical stability, where nearly half of the repeated measurements (49.0%) were identical (ΔHb = 0 g/L). Furthermore, 92.1% of retesting episodes showed a variation of ≤1 g/L, and 98.6% remained within 2 g/L. The overall data analysis showed a satisfying performance of 97.9% of intra-analyser and 80.4% of inter-analyser variation of ≤1 g/L (Figure 1).

**FIGURE 1:**
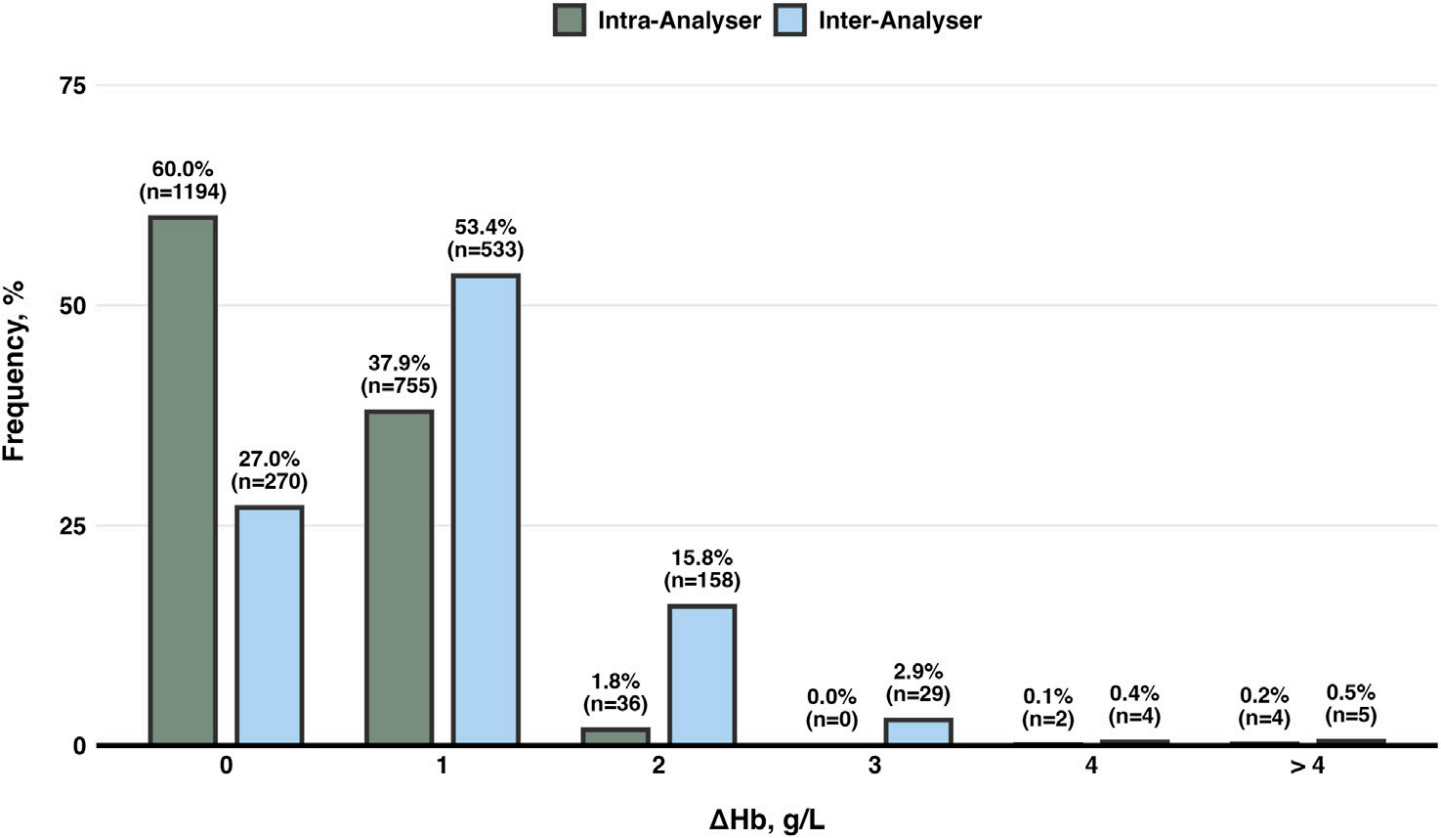
Frequency of maximum haemoglobin variation (ΔHb) between initial and repeated measurements for intra-analyser and inter-analyser comparisons.

Our institution defines Hb <70 g/L as a critical value, triggering further evaluation and notification, but not mandatory transfusion. Transfusion decisions are individualised and approved by Transfusion Medicine physicians in accordance with international guidelines [5]. The 4 g/L threshold is defined as clinically relevant by the Institute for Quality Management in Healthcare (IQMH) [6] and accepted as such in our institution. This value does not impact transfusion decisions or patient management, as signs and symptoms of inadequate tissue oxygenation and hemodynamic status do.

The cut-off of 4 g/L was exceeded in 9 samples, all exhibiting pre-analytical issues (clots, micro-clots, fibrin strands, or mislabelling) that required recollection and were therefore excluded from further statistical analysis.

Baseline analytical performance, mean bias, limits of agreement (LoA), and coefficient of variation (CV%) were calculated from duplicate pairs, as summarised in Supplementary Table S1. For intra-analyser comparisons, mean bias was −0.09 g/L (95% CI: −0.12 to −0.06; LoA: −1.41 to 1.24 g/L; CV: 0.67%), well within the manufacturer’s specified CV of ≤2%. For inter-analyser comparisons, mean bias was −0.19 g/L (95% CI: −0.26 to −0.11; LoA: −2.55 to 2.17 g/L; CV: 1.53%), also within the manufacturer’s inter-analyser specification of ≤4%. The Bland-Altman analysis (Supplementary Figure S1) revealed a small but consistent negative bias for both intra-analyser (−0.09 g/L) and inter-analyser (−0.19 g/L) comparisons, indicating that repeat measurements tend to be marginally lower than initial readings. Both values fall well within the IQMH-defined acceptable range. The wider limits of agreement observed for inter-analyser comparisons (−2.55 to 2.17 g/L vs. −1.41 to 1.24 g/L) reflect the additional variability introduced by between-module differences, which is expected in a multi-analyser platform and consistent with the manufacturer’s inter-analyser specifications. These findings confirm exceptional long-term analytical stability across 5 years of routine 24/7 operation, involving multiple operators and heterogeneous patient samples, and turn unnecessary an automatic non-discriminatory repeat testing of all critical Hb results.

Our analysis did not aim to replace formal precision studies as defined by the Clinical and Laboratory Standards Institute. Instead, it offers a complementary perspective by quantifying real-world performance under routine operational pressures. Whereas controlled experiments define theoretical repeatability and reproducibility limits, longitudinal operational data reflect instrument behaviour among pre-analytical heterogeneity and variable clinical demand.

The primary objective of this study was to evaluate the utility of our SOP mandating reflex repetition of all blood counts with Hb <70 g/L. In doing so, we identified a secondary but equally relevant observation: that systematic review of analyser back-up raw data can serve as a zero-cost internal quality control strategy [7], as these archives contain results from repeat testing triggered by diverse clinical and analytical reasons. Conceptually, such retesting mirrors the classical random-duplicate approach described by Carstairs (1977) [8], in which duplicate measurements were used to evaluate analytical reliability and uncover hidden imprecision or bias [9, 10].

To balance increasing demand with workforce constraints, laboratories must refine workflows through evidence-based strategies. The widespread adoption of data-friendly technologies enables periodic analysis of archived raw back-up, transforming routinely duplicated measurements into continuous performance monitoring tools. In this context, real-world data analytics provide a powerful and sustainable framework to enhance quality assurance and operational resilience in contemporary laboratory practice.

## Data Availability

The raw data supporting the conclusions of this article will be made available by the authors, without undue reservation.

## References

[1] Motie PB Zare-Mirzaie A Shayanfar N Kadivar M . Does routine repeat testing of critical laboratory values improve their accuracy? Clin Lab (2015) 61(11):1731–6. 10.7754/Clin.Lab.2015.150410 PMC4431443 26034729

[2] Sun S-CP Garcia J Hayden JA . Repeating critical hematology and coagulation values wastes resources, lengthens turnaround time, and delays clinical action. Am J Clin Pathol (2018) 149(4):281–6. 10.1093/ajcp/aqx156 29425259

[3] Åsberg A Bolann BJ . Calculating the coefficient of variation from duplicate measurements: a new method. Clin Chim Acta (2023) 538:154–9. 10.1016/j.cca.2022.12.012 36610414

[4] Tosi M Pighi L Rizza M Salvagno GL Lippi G . Intra-and inter-analyzer imprecision of cell population data on sysmex XN-10. eJIFCC (2026) 37(1):159–65. 10.21037/ejifcc-2026-0159 41659287 PMC12882073

[5] Carson JL Stanworth SJ Guyatt G Valentine S Dennis J Bakhtary S Red blood cell transfusion: 2023 AABB international guidelines. JAMA (2023) 330(19):1892–902. 10.1001/jama.2023.12914 37824153

[6] Johnston A Bourner G Martin T McFarlane A Good D Padmore R Guidance for quality control practices and precision goals for CBCs based on IQMH patterns-of-practice survey. Int J Lab Hematol (2019) 41:15–22. 10.1111/ijlh.12915 30138534

[7] McCafferty R Cembrowski G De La Salle B Peng M Urrechaga E . ICSH guidance for internal quality control policy for blood cell counters. Int J Lab Hematol (2024) 46(2):227–3. 10.1111/ijlh.14212 38189640

[8] Carstairs KC Peters E Kuzin EJ . Development and description of the “random duplicates” method of quality control for a hematology laboratory. Am J Clin Pathol (1977) 67(4):379–82. 10.1093/ajcp/67.4.379 851096

[9] Synek V Kříženecká S . Estimation of uncertainty from duplicate measurements: new quantification procedure in the case of concentration-dependent precision. Accredit Qual Assur (2023) 28(6):385–94. 10.1007/s00769-023-01556-9

[10] Cembrowski GS Lunetzky ES Patrick CC Wilson MK . An optimized quality control procedure for hematology analyzers with the use of retained patient specimens. Am J Clin Pathol (1988) 89(2):203–10doi. 10.1093/ajcp/89.2.203 3341279

