# Supplementary material for: Longitudinal agreement of repeated critical haemoglobin measurements as evidence of sustained analytical quality
Source: Br J Biomed Sci. 2026 Aug 3;83:16480. doi: 10.3389/bjbs.2026.16480 (PMC13478016; doi:10.3389/bjbs.2026.16480)
Supplement: Supplementary file 1 [file Supplementaryfile1.docx]

**Figure 1S.** Bland-Altman plots for intra- and inter-analyser agreement of Hb measurements. The y-axis represents the difference between paired Hb measurements and the x-axis the mean of each pair. The solid black line indicates the mean bias; red dashed lines denote the 95% limits of agreement (±1.96 SD). Shaded bands represent the 95% confidence intervals for the bias and limits of agreement. **(A)** Intra-analyser comparison (n = 1987 pairs; bias = −0.09 g/L; LoA: −1.41 to +1.24 g/L). **(B)** Inter-analyser comparison (n = 994 pairs; bias = −0.19 g/L; LoA: −2.55 to +2.17 g/L).
