# Supplementary material for: Longitudinal agreement of repeated critical haemoglobin measurements as evidence of sustained analytical quality
Source: Br J Biomed Sci. 2026 Aug 3;83:16480. doi: 10.3389/bjbs.2026.16480 (PMC13478016; doi:10.3389/bjbs.2026.16480)
Supplement: Supplementary file 2 [file Table1.docx]

**Table S1.** Analytical performance metrics by comparison type. KEY. Mean bias is presented with 95% confidence intervals (CI). Limits of Agreement (LoA = bias ± 1.96 SD) indicate the range within which 95% of individual differences are expected to fall. CV% was calculated per sample pair and averaged within each group.

| Group | N | Mean bias, g/L | 95% CI | LoA, g/L | SD, g/L | CV% |
| --- | --- | --- | --- | --- | --- | --- |
| Global | 2981 | -0.12 | [-0.15, -0.09] | [-1.86, 1.62] | 0.89 | 0.96 |
| Intra-Analyser | 1987 | -0.09 | [-0.12, -0.06] | [-1.41, 1.24] | 0.68 | 0.67 |
| Inter-Analyser | 994 | -0.19 | [-0.26, -0.11] | [-2.55, 2.17] | 1.20 | 1.53 |
